# A Review on Recent Progress of Stingless Bee Honey and Its Hydrogel-Based Compound for Wound Care Management

**DOI:** 10.3390/molecules27103080

**Published:** 2022-05-11

**Authors:** Nur Eszaty Farain Esa, Mohamed Nainar Mohamed Ansari, Saiful Izwan Abd Razak, Norjihada Izzah Ismail, Norhana Jusoh, Nurliyana Ahmad Zawawi, Mohamad Ikhwan Jamaludin, Suresh Sagadevan, Nadirul Hasraf Mat Nayan

**Affiliations:** 1 Bioinspired Device and Tissue Engineering Research Group, Faculty of Engineering, School of Biomedical Engineering and Health Sciences, Universiti Teknologi Malaysia, Skudai 81300, Johor, Malaysia; nefarain2@graduate.utm.my (N.E.F.E.); norjihada@utm.my (N.I.I.); norhana@biomedical.utm.my (N.J.); nurliyana@utm.my (N.A.Z.); mohd.ikhwan@utm.my (M.I.J.); 2 Institute of Power Engineering, Universiti Tenaga Nasional, Kajang 43000, Selangor, Malaysia; 3 Medical Device Technology Center (MEDiTEC), Institute Human Centred Engineering (iHumEn), Universiti Teknologi Malaysia, Skudai 81310, Johor, Malaysia; 4 Nanotechnology & Catalysis Research Centre, University of Malaya, Kuala Lumpur 50603, Kuala Lumpur, Malaysia; drsureshnano@gmail.com; 5 Faculty of Engineering Technology, Universiti Tun Hussein Onn Malaysia, Batu Pahat, Bahru 86400, Johor, Malaysia; nadirul@uthm.edu.my

**Keywords:** stingless bee honey, hydrogel, wound healing, antioxidants, antibacterial, anti-inflammatory, natural moisturizer

## Abstract

Stingless bee honey has a distinctive flavor and sour taste compared to *Apis mellifera* honey. Currently, interest in farming stingless bees is growing among rural residents to meet the high demand for raw honey and honey-based products. Several studies on stingless bee honey have revealed various therapeutic properties for wound healing applications. These include antioxidant, antibacterial, anti-inflammatory, and moisturizing properties related to wound healing. The development of stingless bee honey for wound healing applications, such as incorporation into hydrogels, has attracted researchers worldwide. As a result, the effectiveness of stingless bee honey against wound infections can be improved in the future to optimize healing rates. This paper reviewed the physicochemical and therapeutic properties of stingless bee honey and its efficacy in treating wound infection, as well as the incorporation of stingless bee honey into hydrogels for optimized wound dressing.

## 1. Introduction

Wound healing is one of the most challenging physical processes and requires a great deal of attention [1,2]. Hemostasis, inflammation, proliferation, and maturation are all phases of wound healing physiology. In the first phase, hemostasis, the platelets and fibrin clump together to form a thrombus, plugging broken blood arteries and preventing blood loss. The inflammatory phase then begins, during which a large number of neutrophils and macrophages enter the wound area to engulf and destroy bacteria, pathogens, or debris via the phagocytosis process. During the proliferation phase, angiogenesis begins, and granulation tissue is produced to replace injured tissue. This is followed by the maturation phase, which begins three weeks after the injury event and lasts more than a year. The wound area becomes rich in collagen and other extracellular matrix deposition (ECM) proteins over time [1,2,3].

Non-healing wounds, on the other hand, are wounds that do not heal in a timely and orderly manner and can cost up to USD 50 billion in the United States alone, while scars from surgical incisions and trauma cost roughly USD 12 billion, and burns cost USD 7.5 billion in healthcare each year [4]. Clinical trials have shown that the dressing must continue to function as a temporary barrier to prevent infection and fluid loss [5]. Hydrogel technology, which recognizes the significance of the wound healing process, offers suitable products for biomedical applications, particularly in wound healing. Hydrogels are recommended for wounds that are dry to mildly exuding; they have a noticeable cooling and soothing effect on the skin and are beneficial for burns and painful wounds [3]. They are also effective in softening necrotic tissue on the wound surface and hydrating wound surfaces; they can be used or removed without interfering with the wound bed and they are non-adherent [6,7]. Thus, there is much interest in developing hydrogel-based wound dressings as they appear to be the best media for improving wound healing.

Stingless bee honey has been shown to have therapeutic properties, such as antioxidant, antibacterial, anti-inflammatory properties, and is a natural moisturizer for wound healing applications. It has the potential to be commercialized in the pharmaceutical industry. The high number of polyphenol compounds in stingless bee honey mean it is good at promoting cell proliferation, protecting the cellular structure, and fighting against free radicals at the wounded area [8,9,10,11]. Moreover, the antibacterial properties of stingless bee honey can inhibit bacteria and pathogenic organisms at the wounded area and accelerate wound healing via epithelization [12] due to its low pH and high free acidity [13,14]. Furthermore, clinical trials have shown that honey-based hydrogels are able to improve water absorption and swellability, have high antibacterial properties, support epithelialization, enhance cell proliferation, inhibit bacteria growth, and shorten the wound healing process [15,16]. The same goes for stingless bee honey-based hydrogels, which have shown significant benefits such as preventing wound infection, providing an excellent moisturizing environment, and encouraging cell viability at the wounded area [17,18].

## 2. Stingless Bee Honey Properties and Benefits

Stingless bee honey is known for its medicinal properties in healing wounds. Many researchers have studied the physicochemical properties of stingless bee honey, which aid in wound healing and are comparable to *Apis mellifera* honey properties. Stingless bee honey is a highly effective antioxidant [14,19,20], antibacterial [10,20,21,22,23,24], and anti-inflammatory agent [10,20,25]. This honey also can act as a moisturizer for wounds [10,14,26,27]. Because of its extraordinary healing properties, various studies on stingless bee honey have been developed to investigate and evaluate its efficacy in treating wounds. These studies are still in the early phases of development, and stingless bee honey has not yet received enough clinical testing.

### 2.1. Stingless Bee Honey

Stingless bee honey is a product obtained from plant nectar and exudate or from the excretion of plant-sucking insects. As the popular name suggests, stingless bees are so-called as they do not have a sting unlike the *Apis mellifera* bees, which produce traditional honey and are recognized worldwide [28,29]. These bees are divided into two tribes. The first is Meliponini, which only consists of the genus *Melipona*; they are found exclusively in the Neotropics. The other tribe, Trigonini, is found throughout the tropics [20]. Stingless bees live in tropical and subtropical areas around the world such as Malaysia, Brazil, Mexico, Africa, Northern Australia, and the Southeast Asian regions [30]. Stingless bees are native to Africa [30,31]. In tropical countries, such as Malaysia, Thailand, Mexico, Venezuela, Brazil, and Australia, beekeeping using stingless bees is a well-known tradition [13,32].

**Figure 1:**
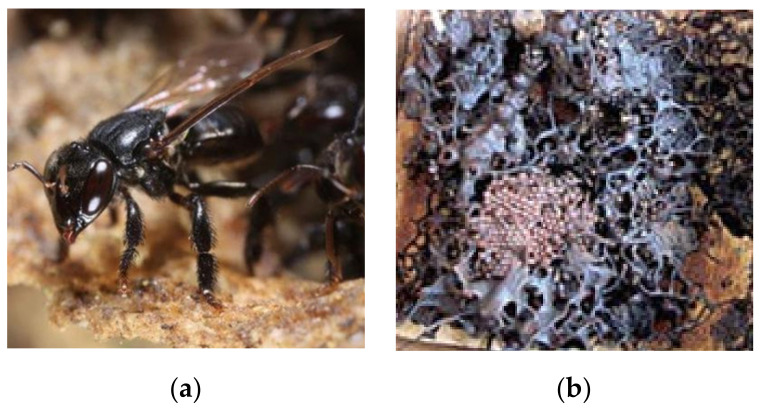
(**a**) Stingless bee honey; (**b**) hive containing stingless bee honey.

When it comes to living conditions, stingless bees choose habitats that are safe and conducive to their existence. Physical environmental elements have an impact on their hive’s micro-climate. Furthermore, they enjoy warm weather and are more active in the sun than in cold or gloomy conditions [13,31]. The stingless bee’s distinctive features include collecting nectar from creeping plants, flying short distances searching for food, and developing hives positioned horizontally instead of vertically to store nectar and pollen. Stingless bees produce honey and other bee by-products, such as pollen, beeswax, cerumen, and royal jelly. These bees store their honey in cerumen, a mixture similar to propolis but with the addition of the mandibular secretions produced during construction by the stingless bee’s mandibles [10,13,33]. The production of stinglees bee honey is well known for its distinct flavor and aroma, as well as its fluid texture and slow crystallization [20,34,35]. However, it lacks identity and quality requirements due to a lack of understanding of its composition [36]. Therefore, the empowerment of today’s stingless bee sector would directly impact the production of high-quality honey while also supporting the pollination of crops and other plants, and thus conserving biodiversity [30]. Figure 1 below shows the stingless bee and a hive where the honey is stored.

### 2.2. Physicochemical Properties of Stingless Bee Honey

**Figure 2:**
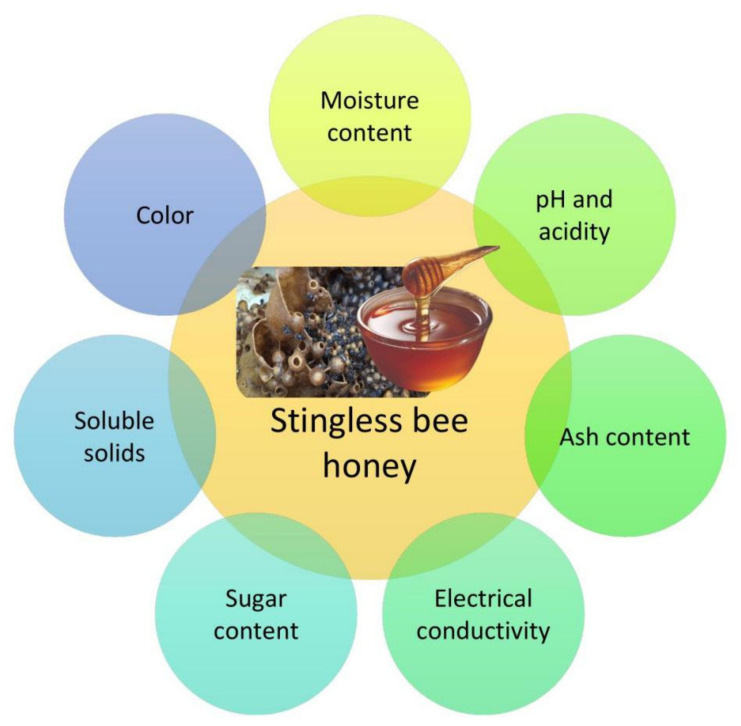
The important parameters for physicochemical properties of stingless bee honey.

Stingless bee honey differs from that of the genus *Apis* in terms of color, taste, and viscosity. Honey from this species was shown to have higher free acidity and moisture levels than *Apis mellifera* honey but lower pH and total soluble solids than existing standards [37]. The proteins, amino acids, enzymes, organic acids, mineral elements, and vitamins found in honey are relatively small compared to the other components [20,26,36]. However, the availability of specific elements in honey is determined by various parameters, including floral source, bee type, geographical origin, weather, harvesting season, processing method, and storage conditions [13,26]. Different species of bees have distinct physicochemical features and generate different qualities of honey. Figure 2 below shows the important parameters for the physicochemical properties of stingless bee honey.

The moisture level is a significant factor in determining honey quality because high moisture content can lower a honey’s shelf life and microbiological stability. A high moisture level is related to the honey’s water content, which affects viscosity, specific weight, maturation, crystallization, and flavor [14,38,39,40]. Stingless bee honey has been reported to contain greater water content than *Apis mellifera* honey [13,14,41]. This is owing to the humid, tropical nature of their habitat, difficulty in extracting nectar with low water content, and other considerations such as the fact that stingless bees gather nectar from undergrowth flowers and ripe fruits that are rich in water. The high water content makes it easier for microorganisms to survive and be active, making fermentation more likely and making preservation and storage more complicated [20,26,42,43].

According to Kek et al. [14], stingless bee honey has a lower pH and a higher free acidity than *Apis mellifera* honey [13,41]. The pH value of stingless bee honey is influenced by various factors, including extraction and storage processes. Additionally, the pH of honey can alter its texture, stability, and shelf life. The majority of bacteria thrive in a neutral or slightly alkaline environment. This property is a highly accurate predictor of microbial stability. In addition, the acidity content enhances the flavor of stingless bee honey [26,38,39]. High acidity may suggest that sugars have been fermented into organic acids [13], increasing free acidity. The presence of organic acids alters the free acidity value of stingless bee honey.

The ash content of honey correlates to its mineral content and is influenced by the geographical, environmental, and botanical characteristics of the honey’s origin [44]. Stingless bee honey has been reported to possess higher ash content than *Apis mellifera* [13,14,21,45,46]. The variations in ash content in honey may be related to the material collected by bees while foraging on flora, to the non-uniformity of harvesting and beekeeping procedures, and also to the fact that different varieties of honey come from different botanical sources [14,26,39]. Moreover, the electrical conductivity of the honey is directly related to its concentration of minerals, salts, organic acids, and proteins, and it is a parameter that shows great variability depending on the floral source of honey [13,20,40,47]. Stingless bee honey from Brazil has lower electrical conductivity compared to *Apis mellifera* honey [20], while Thailand’s stingless bee honey has a higher value of electrical conductivity compared to *Apis mellifera* honey from Thailand [46,48].

Stingless bee honey has less sugar content than *Apis* *mellifera* honey [40]. Fructose and glucose, two reducing sugars, are generally considered to account for the majority of honey’s sugar content [47]. Additionally, honey also contains sucrose and maltose, which are generally reported at lower concentrations than fructose and glucose or not detected at all [47,49]. However, stingless bee honey contains more sucrose and maltose than regular honey [13]. Therefore, this low sugar level and acidic pH would affect the flavor of stingless bee honey [40]. Aside from that, the soluble solids in honey include sugars, organic acids, and minerals. Due to the fact stingless bee honey contains more water and less sugar than *Apis* *mellifera* honey, the soluble solid value is lower in stingless bee honey [13,20,36].

The color of honey is determined by its mineral concentration, pollen content, phenolic content, duration of storage, and temperature [14,47]. Honey’s color varies considerably depending on its geographical origin. Correspondingly, production and agricultural methods can affect the color of honey [13,41]. The color of stingless bee honey has been documented to range from light amber to amber brown, and those with darker colors have greater ash values than those with lighter colors [40,49,50]. This characteristic may also be affected by factors such as light exposure, heat, and duration of storage, as well as enzymatic reactions [20,49]. The darker-colored honey contains a higher quantity of total phenolics, indicating that it may have a high level of bioactivity [44].

### 2.3. Therapeutic Properties of Stingless Bee Honey

The use of honey as a remedy to treat wounds is well known around the world. Honey is commonly used as an antioxidant, antimicrobial, and anti-inflammatory agent [11,15]. This is because honey is highly nutrient-dense and includes trace minerals and vitamins. The remarkable healing properties of honey have been used to treat septic wounds, surgical wounds, and wounds of the abdominal wall and perineum [51]. Stingless bee honey, in particular, it has been claimed, possesses medicinal characteristics and provides various health advantages when applied to wounds. The number of studies examining this honey’s therapeutic benefits and physicochemical aspects is increasing, and these benefits are attracting considerable interest. The high price of stingless bee honey is due to the high levels of flavonoids and polyphenols present in stingless bee honey compared to *Apis mellifera* honey [52]. Stingless bee honey has antioxidant, antibacterial, anti-inflammatory, and natural moisturizing properties that aid in wound healing. Moisture content, water activity, pH, peroxide, non-peroxide, phenolic acids, flavonoids, vitamins, and enzymes are likely to be the components of stingless bee honey that contribute to fast wound healing [10].

#### 2.3.1. Antioxidant Activity

Antioxidants are compounds that can donate an electron to free radicals, neutralizing, reducing, or eliminating their ability to damage cells and essential biomolecules such as nucleic acids, proteins, and lipids [8]. Antioxidants protect the cellular structure by neutralizing reactive oxygen species (ROS). They can prevent other molecules from oxidizing. Oxidation is a biological phenomenon that produces free radicals, which can cause damage to cells, tissues, and eventually physiological functioning. Antioxidants can block chain reactions, thereby protecting the cell from free radicals [10,11,53]. The potent antioxidant capacity of honey is a result of the forest’s biodiversity. This is due to the high concentration of phenolic and flavonoid compounds, which boosts antioxidant activity [54].

The primary antioxidants are phenolic acids and flavonoids, the most abundant polyphenols in this beehive product [39,55]. Stingless bee honey contains a higher level of phenolic acids and flavonoids than honey produced by *Apis* *mellifera* [20]. This phenolic compound stabilizes free radicals by releasing hydrogen from one of their hydroxyl groups [8]. The stingless bee honey in Brazil has greater phenolic content than *Apis* spp., with 106.01 ± 9.85 mg GAE/100 g as compared with 92.34 ± 13.55 mg GAE/100 g for *Apis* spp. [56]. Similarly, stingless bee honey contains around 235 mg GAE/100 mg of total phenolic content in Malaysia compared to Tualang honey from *Apis* spp., which contains 183 mg GAE/100 mg, while the flavonoid content of stingless bee honey is 100 mg CE equivalent 100 mg [57,58]. Stingless bee honey from Cuba was also shown to have stronger antioxidant activity and a higher content of flavonoids, carotenoids, ascorbic acid, phenolic compounds, free amino acids, and protein compared to *Apis* *mellifera* honey [59]. The antioxidant activity of stingless bee honey produced by *Heterotrigona itama* bees was double that of *Apis* *mellifera* honey, according to a study by Kek et al. [14].

Both antioxidant enzymes and non-enzymatic compounds are present in stingless bee honey, where antioxidant enzymes such as superoxide dismutase neutralize the free radical or reactive oxygen species into a molecule that is less harmful to the body. In contrast, non-enzymatic compounds such as ascorbic acid, tocopherol, and phenolic compounds neutralize reactive oxygen species by blocking and cutting damaging chain reactions caused by free radicals [19]. The high phenolic and flavonoid content in stingless bee honey samples is due to polyphenol compounds such as gallic acid, chrysin, caffeic acid, apigenin, kaempferol p-coumaric acid, 4-hydroxybenzoic acid, and quercetin-3-O-rutinosoid, which have antioxidant properties [60]. Moreover, a study by Biluca et al. [61] proved the presence of phenolic compounds in stingless bee honey (*Meliponinae*). The compounds are mandelic acid, caffeic acid, chlorogenic acid, rosmarinic acid, aromadendrin, isoquercitrin, eriodictyol, vanillin, umbelliferone, syringaldehyde, sinapaldehyde, and carnosol. These compounds contribute to the high level of antioxidant activity of stingless bee honey [61].

**Figure 3:**
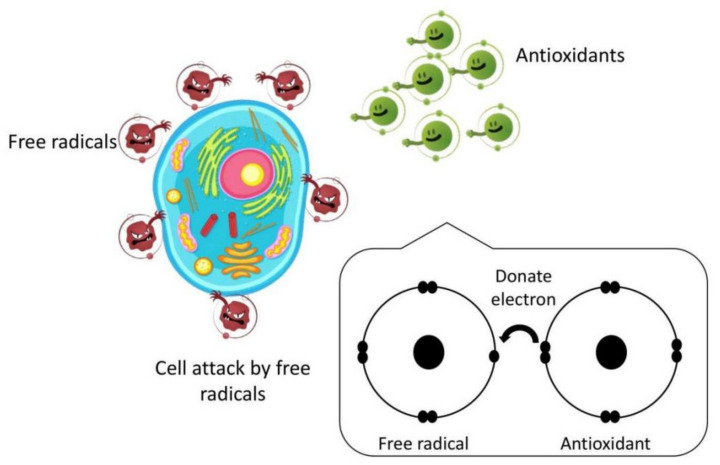
Illustrations of antioxidants fighting against free radicals by donating electrons.

Polyphenol substances present in stingless bee honey contribute to the honey’s appearance and functional properties [10,34]. Malaysian *Trigona* honey possesses higher total phenolic content compared to Malaysian *Apis* honey [21]. According to Mohamad et al. [9], the polyphenol components in stingless bee honey generated by *Trigona bees* enhance cell growth. Due to the fact that phenolic compounds are derived from plants, the phenolic and flavonoid content of honey is incredibly reliant on the nectar source selected by the bees, the bee species, and the bees’ foraging region. Honey’s phenolic content is also affected by factors such as harvest season, weather, and processing conditions [26,54,62]. As a result, stingless bee honey has a high antioxidant capacity, implying potential health advantages. The phenolic and flavonoid content of stingless bee honey acts as a powerful antioxidant, scavenging free radicals and resulting in more stable and less reactive radical species. Figure 3 shows the illustrations of antioxidants fighting against free radicals by donating electrons.

#### 2.3.2. Antibacterial Agent

Currently, numerous products use honey as a cure for a variety of diseases including bacterial infections. Wound healing is inextricably linked to antibacterial activity [63]. Microorganisms are becoming less sensitive to drugs, making them more resistant to a wide variety of medications. This could result in a significant emerging problem known as multidrug resistance. Only a few antimicrobial drugs are available, which are typically expensive and can have various adverse effects on the body [64]. The antibacterial compounds of honey are effective against many harmful microorganisms [65,66]. Current research on stingless bee honey has demonstrated that it is efficient against many harmful microorganisms without any adverse effects [19].

Stingless bee honey containing antibacterial compounds is effective against a broad spectrum of harmful microorganisms. Its antibacterial properties are due to several variables, including hydrogen peroxide, a low pH, and a high osmolarity [24]. It can aid in healing wounds, skin ulcers, burns, and inflammations and in promoting new tissue to heal lesions [8,53,63]. It can also aid in sterilizing wounds and in reducing the edema and scarring that result from superficial wounds, diabetic foot ulcers, and pressure ulcers [8]. The variation in the antibacterial capacity of honey, even when produced by similar bee species, could result from various factors. The factors include the fact of the honey being collected in different geographical locations, seasonal conditions, flower source, processing, storage conditions, and the bacteria’s sensitivity to antibacterial compounds in honey [67].

The antibacterial properties of stingless bee honey can be classified into peroxide and non-peroxide [10]. The majority of the antibacterial action has been attributed to hydrogen peroxide activity. One of the enzymes contained in honey is glucose oxidase, which transforms glucose into gluconolactone and eventually into hydrogen peroxide. Hydrogen peroxide breakdown produces highly reactive free radicals that react with and destroy microbes [22,68]. The hydrogen peroxide production is slow and continuous, resulting in a continual antibacterial activity that effectively eliminates germs while diluting sufficiently to avoid injury to host tissue [10,12]. It can, however, be quickly terminated in the presence of heat or catalase activity [10,22].

The antibacterial activity of stingless bee honey, which is more consistent and stable than the antibacterial action of *Apis mellifera* honey, may also result from non-peroxide activity [20,22]. The non-peroxide components of honey are derived from phytochemicals, sugar content, and acidity. The phytochemical features of honey include flavonoids, phenolic acids, and antimicrobial peptides. Flavonoid compounds are reported to be responsible for antibacterial properties, and the flavonoid compounds present in stingless bee honey are naringenin, kaempferol, apigenin, pinocembrin, and chrysin. For phenolic acids, the types of compounds present in stingless bee honey are protocatequic acid, *p-* hydroxibenzoic acid, caffeic acid, chlorogenic acid, vanillic acid, *p*-coumaric acid, benzoic acid, ellagic acid, and cinnamic acid [69]. These components aid in inhibiting bacteria growth, prevent superoxide free radicals from causing tissue damage, and also speed up wound healing [10,19,70]. The antimicrobial peptides were found in Manuka honey [71]; however, Ng et al. [69] reported that the stingless bee honey did not contain methylglyoxal (MGO), dihydroxyacetone, or phenolics characteristic of manuka plant nectars. Therefore, they consider phenolic and flavonoid compounds to be the only phytochemicals that contributed to the non-peroxide antibacterial activity of stingless bee honey [69].

Another factor is high osmolarity, which is due to the high sugar content of stingless bee honey; this can also inhibit the growth of bacteria, which are highly attracted to water molecules, by leaving them with inadequate water to survive [12,22,63,72]. Moreover, stingless bee honey, which has a lower pH and a higher free acidity [13,14], inhibits a wide variety of pathogenic organisms and accelerates wound healing via epithelization [12]. The acidity environment alters the metabolism of bacteria by interfering with enzymatic activities and disrupting plasma membrane integrity [69].

Moreover, the high concentration of honey affects antibacterial effectiveness [73]. When used as a dressing, honey solutions are diluted by wound exudate, which reduces their osmolarity and thus blocks the honey solution’s capacity to control infection; this mainly occurs when wounds are infected with *S. aureus,* a common osmotolerant wound pathogen. Even when honey is diluted with the exudate to the point where its osmolarity no longer prevents bacterial development, the extra antibacterial components in honey preserve sterility [12,63]. Moreover, the strongly acidic environment of stingless bee honey may partly explain why it possesses such potent antibacterial capabilities and may be lethal to a variety of bacteria [66,70,74,75]. Additionally, stingless bee honey can also shorten the infection time for eye diseases caused by *S. aureus* and *P. aeruginosa* and protect against gastrointestinal infection in humans [76].

#### 2.3.3. Anti-Inflammatory

Inflammation is a natural physiological response that occurs in the body in response to infections, tissue damage, or irritations. It can produce soreness, edema, and exudation, and it can slow or restrict the healing process [77]. Under normal circumstances, the inflammatory response removes infectious organisms from the body and aids in tissue repair [78]. However, prolonged inflammation results in a progressive shift in the cell type present at the inflammatory site and is characterized by the simultaneous destruction and repair of the inflammatory tissue [78,79,80]. As a result, pathological diseases such as autoimmune disease, tissue fibrosis, or tumor growth may develop [78]. Numerous anti-inflammatory medications have been produced; however, they are incompatible with wound healing. For instance, non-steroidal anti-inflammatory medicines (NSAIDs) are cytotoxic to tissues, while corticosteroids can hinder epithelial tissue development. Consequently, wound care products include minimal anti-inflammatory activity, although inflammation remains the primary cause of chronic wound healing delays [10].

The introduction of honey for wound care is an excellent way to reduce inflammation. Stingless bee honey contains anti-inflammatory agents, and it shows anti-inflammatory effects when applied to the skin. The anti-inflammatory properties of stingless bee honey contribute to less scarring during the healing process of burn wounds. This activity may be triggered by the phenolic components in stingless bee honey that act synergistically [25]. The synergistic action of the sugar and hydrogen peroxide in stingless bee honey, which work in concert with each other, contributes to wound healing [81]. Phenolic acid such as p-coumaric acid, caffeic acid, and salicylic acid taken from an extract of stingless bee honey for one study also decreased ROS production at the wounded area [76]. Flavonoids compounds such as kaempferol, quercetin, luteolin, genistein, apigenin, galangin, naringenin were able to reduce the expression of different pro-inflammatory cytokines/chemokines [82]. They operated as free radical scavengers, shielding cells from the cytotoxicity caused by proinflammatory mediators. The duration of inflammation during the wound healing stage was reduced, which increased the healing rate [10].

The extraction of stingless bee honey possesses anti-inflammatory activity which reduces ear edema, most likely due to the synergistic effect of its phenolic compounds such as kaempferol and caffeic acid. It also suppresses myeloperoxidase activity, leukocyte infiltration, and the production of reactive oxygen species (ROS) [25,83]. Another study also reported the presence of p-coumaric acid, salicylic acid, aromadendrin, and taxifolin in stingless bee honey, and these compounds contributed to ameliorating acetaminophen-induced hepatic inflammation by regulating the pro-inflammatory cytokines IL-1β, IL-6, and TNF-α [32,84].

Furthermore, the harvesting area of stingless bee honey affects its anti-inflammatory properties. Stingless bee honey extracts from different states in Malaysia show different anti-inflammatory activity towards inhibiting nitric oxide production [85]. This implies that the pharmacological characteristics of stingless bee honey are location dependent. Further, stingless bee honey has a non-toxic effect on dermal fibroblasts, suggesting the possibility of applying stingless bee honey to treat wound healing [13]. Additionally, El-Kased et al. [86] stated that no apparent infection occurred at the wound site after application. The stingless bee honey continued to reduce inflammation, indicating that honey’s anti-inflammatory activity is a direct effect, not a side effect of the antibacterial activity that eliminates infection.

#### 2.3.4. Natural Moisturizer for Wounds

Numerous research studies have demonstrated that stingless bee honey contains a significant level of moisture [13,14,38,39,41]. Honey has significant moisturizing properties due to the presence of hydroxyl groups. Sugars, proteins, and lactic acid are all components of honey that function as moisturizers. Additionally, vitamins B, E, and K and a variety of essential minerals such as potassium, phosphorus, and calcium contribute to the moisturizing characteristics of stingless bee honey [10,27]. The high water content of stingless bee honey helps prevent wound dryness through the osmotic effect, which gradually retains fluid within the wound tissue [14,26,38]. This process can retain a moist environment around the wound tissue and, as previously said, will promote healing. However, a continually humid environment can deteriorate into wet conditions, resulting in wound maceration. Fortunately, the honey’s high osmolarity protects the skin against maceration and helps to maintain a moist environment [10]. Since osmosis forms a honey solution in contact with the wound surface, which keeps the dressing from sticking, dressing changes are painless and cause no tissue damage [87]. Thus, this shows that the moisturizing characteristics of stingless bee honey are crucial for a patient’s speedy recovery.

### 2.4. Evaluation of Stingless Bee Honey for Wound Healing

As previously mentioned, stingless bee honey possesses therapeutic agents that can help in the healing of wounds. Researchers around the world have developed and evaluated the efficacy of stingless bee honey as a wound infection treatment. A study conducted by Ng et al. [88] evaluated stingless bee honey’s inhibitory activity against *S. aureus* planktonic and clinical biofilm cultures and showed antibacterial action against both antibiotic-sensitive and antibiotic-resistant *S. aureus.* This indicates that the phenolic compounds, acidity, and hyperosmolarity of the stingless bee honey contribute to the excellent inhibitory effect. In another study, the stingless bee honey appeared to successfully prevent essential colonization development in a diabetic foot ulcer wound infection. This was due to its antibacterial and antioxidant characteristics. The higher water content of stingless bee honey also reduced the pain, while the lower pH amplified its antibacterial activity, making it ideal for wound treatment. As a result, the wounds had a shorter inflammatory phase, were odorless, and healed more quickly [89].

Furthermore, using a low dose of stingless bee honey in treating wounds enhances the growth and proliferation of cutaneous fibroblasts. In another study, honey increased proliferation in dermal fibroblasts and did not affect normal cell cycle progression. In addition, the long-term effect of low-dose honey on dermal fibroblast proliferation revealed that honey had a continuous beneficial impact during the proliferative phase of wound healing. However, these authors noted that a greater dose of stingless bee honey also enhances cutaneous fibroblast survival after 24 h of exposure but fibroblast proliferation is slow [90].

A study on the effect of stingless bee honey on TGF-induced epithelial to mesenchymal transition (EMT) in human primary keratinocytes in the context of scar-free wound healing also stated that a low dose of stingless bee honey demonstrated high cell viability and non-toxicity in epidermal keratinocytes and led to a more directional migration. The stingless bee honey therapy was able to reverse the TGF-induced increase in wound closure. This shows that stingless bee honey has medicinal potential in preventing excessive wound healing. It can also prevent over-induction of wound healing; thus, it can be utilized as an anti-keloid agent. This study indicates that honey promotes keratinocyte migration while preserving epithelial integrity and can be used in scar-less wound healing applications [91].

Kirupha et al. [70] developed a nanofibrous composite membrane for wound healing by combining stingless bee honey from India and curcumin (turmeric). The results indicate that the combination of curcumin and stingless bee honey improves wound healing by promoting skin cell migration and enhances recovery via its anti-inflammatory characteristics. The combination of stingless bee honey and curcumin aids in the debridement of dead tissue in the wound bed, prevents scar formation, and encourages regeneration. When compared to untreated control groups, the results of in vitro and in vivo wound models were enhanced recovery and no cytotoxicity. Additionally, the antioxidant and antibacterial findings indicated the wound healing activity, with the membrane demonstrating better activity when compared to curcumin and honey alone. As a result, the inclusion of stingless bee honey from India in this composite membrane can aid wound healing.

## 3. Available Wound Dressings for Treating Wounds

Wound dressing is used to repair the damage produced by an abrasive stimulation in order to shield the wound from the outside environment, promote healing, and minimize the risk of infection [92]. Persistent infections and the incapacity of dermal and epidermal cells to respond to reparative stimuli are some of the characteristics that contribute to the failure of wounds to heal [93,94]. Disease, pharmacological therapy, and the patient’s circumstances must be assessed and addressed before using a specific wound dressing [2]. The optimal dressing materials should supply or maintain a moist wound environment, absorb excess exudate, and avoid maceration of surrounding skin and desiccation of the wound. They should provide thermal insulation to keep the wound core temperature stable and function as a barrier to germs, thus reducing wound infection [2,95]. Advanced wound treatment strategies should also include non-invasive healing monitoring, pain management, and the controlled release of medications capable of stimulating regeneration, repair, and scar reduction [95]. The materials employed should also be cost-effective and require infrequent dressing changes [3].

Wounds are divided into acute and chronic based on their healing physiology and characteristics. Different types of dressings are used in treating acute and chronic wounds. An acute wound is a skin injury that occurs abruptly rather than gradually. It heals at the standard wound healing rate, which is predictable and expected. It can occur anywhere on the body and ranges in severity from minor scratches to deep wounds that damage blood vessels, nerves, and muscles. Burns, other traumatic injuries, and surgically created wounds are examples of acute wounds that heal quickly [96]. The available dressings for treating acute wounds include a conventional gauze dressing, a nonadhesive dressing (silicone or paraffin gauze), hydrocolloid dressing, and film dressing [97,98,99]. Moreover, hydrogel-based dressing is already used for burns as it can absorb excess exudates, give a cooling effect, and keep a moist environment at the wound site [100].

Meanwhile, chronic wounds are wounds that have failed to heal in a timely and orderly manner and have become a severe burden to healthcare systems globally [93,101,102]. They are most commonly associated with diabetes mellitus, venous stasis, peripheral vascular disorders, and pressure ulcers, and they remain inflamed indefinitely, which is the second stage of wound healing [93,103,104,105]. There are several treatments available for chronic wounds, which are antibiotics, negative pressure wound therapy (NPWT), and skin grafts. However, these treatments have their own disadvantages: antibiotics do not promote granulation of the ulcer [94] and NPWT can cause skin irritation, painful dressing changes, entero-atmospheric fistulae, and the risk of bleeding in patients using anticoagulants [106,107], while skin graft causes skin pigmentation and skin graft contraction [108,109]. At the same time, modern wound dressings are also available such as hydrocolloid dressing, alginate dressing, foam dressing, and hydrogel dressings to treat chronic wounds.

Hydrocolloid dressings can attach to both wet and dry locations, provide a shorter healing time, lower frequency of infections, and minimal pain, and are cost-effective dressings [2,99,110]. Granuflex™ and Comfeel™ are available hydrocolloid dressing in the form of sheets or thin films. Nevertheless, the drawbacks are that they are not recommended for neuropathic ulcers or excessively exuding wounds and that they are used primarily for secondary dressings [2,97]. Alginate dressings are used because they can form gels when in contact with wound exudates. This results in excellent absorbency, and helps alginate dressings to maintain a physiologically moist environment while minimizing bacterial infections at the wound site [2,111]. Commercially available alginate dressings include Algicell^TM^ and AlgiSite M^TM^, which are used to treat diabetic foot ulcers, leg ulcers, pressure ulcers, and traumatic and surgical wounds [111]. However, alginate dressings need extra dressings since they can dry the wound, thus delaying recovery. As such, these dressings are not recommended for dry wounds, wounds with third-degree burns, or severe wounds with exposed bone [97]. Next, foam dressings can maintain wound moisture, provide thermal insulation, and are comfortable to wear. They are highly absorbent, and their absorbency is determined by the texture, thickness, and pore size of the foam. Foam dressings are also used for granular wounds, and have been shown to help with overgranulation treatment. However, this dressing does not fit well around the curves of the hands [2,112].

Hydrogel dressings are utilized to heal severe wounds. Hydrogel dressings are ideal for healing dry and necrotic wounds due to the high water content of hydrogels [100]. Additionally, hydrogels have a cooling and relaxing effect and help relieve the pain associated with dressing changes [95]. Hydrogel dressings are used to treat chronic leg ulcers, pressure ulcers, and burns [2,97]. Intrasite^TM^, Nu-gel^TM^, AquaformTM polymers, sheet dressings, impregnated gauze, and water-based gels are all examples of hydrogels. Nonetheless, this form of dressing can accumulate exudate, resulting in maceration and bacterial development and producing a foul odor in wounds. It also has a low mechanical strength, making it difficult to handle [97]. Therefore, the types of dressings and treatments used usually depend on the types of wounds. It is essential to consider each wound individually in order to create the optimal conditions for wound healing. Doctors and physicians choose the best treatments and wound dressings for each wounds to ensure the wound heals quickly without any infection.

## 4. Hydrogel-Loaded Honey as an Ideal Wound Dressing

**Figure 4:**
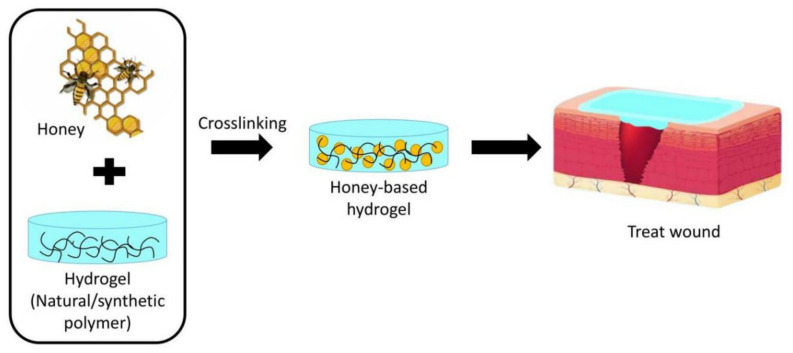
Schematic of honey-based hydrogel.

Hydrogel and honey have many advantages and should be considered an ideal wound dressing to enhance the wound healing rate. Hydrogels have excellent properties that would promotes wound healing. However, single-component hydrogels have a poor mechanical strength [113], and hydrogel-loaded drug dressings are not cost-effective, pose safety concerns regarding the use of drugs, and need to make clear whether they contain allogeneic or xenogeneic materials for patient’s safety [100]. Thus, new advances have introduced composite or hybrid hydrogels to meet conventional wound dressing needs, and the use of honey incorporated into hydrogels is being studied and evaluated. Most of studies have reported that it could promote wound healing better. The combination of both has the potential to be an ideal wound dressing. Figure 4 shows a schematic of a honey-based hydrogel.

### 4.1. Hydrogels

Hydrogels are high-water-content materials made from cross-linked polymers. They are a significant class of biomaterial in biotechnology and medicine due to their excellent biocompatibility and their induction of minimal inflammatory responses, thrombosis, or tissue damage [114]. Hydrogels also can mimic human tissue’s extracellular matrix (ECM) and encourage cell proliferation and migration; they are able to release drugs or growth factors, they cause minimal mechanical irritation to surrounding tissue, and they promote nutrient diffusion, all of which contribute to cell viability and proliferation [86,115]. Hydrogels provide a platform for the loading of cells, antibacterial agents, and growth factors [116], as well as a way of initiating the wound repair process [117]. The critical means by which hydrogels stimulate wound healing is by transferring moisture between the wound bed and the dressing, producing an ideal microenvironment. Due to their high moisture content, these dressings also provide a cooling and calming effect, which helps patients comply with dressing changes and relieves the pain associated with them [6,95].

Hydrogels made from various natural and synthetic polymers have been extensively researched for numerous treatments with varying mechanical strengths and biological reactions. Polymeric wound dressings based on hydrogels accelerate pressure ulcer healing by encouraging epithelialization. Thus, 85% of lesions heal with hydrogel dressings, compared to 50% with standard gauze dressings [113,118,119]. Hydrogels based on natural saccharides are promising for wound healing applications as they possesses good biocompatibility and have low toxicity, immunogenicity, and susceptibility to enzymatic breakdown [86,120]. Polysaccharide is a type of natural polymer that has been employed as a structural material in hydrogels such as chitosan, alginate, and cellulose.

Chitosan-based hydrogels have excellent bioadhesive, bacteriostatic, and hemostatic characteristics, including the capacity to bind with red blood cells and cause blood to clot [105], as well as to act as potential scaffolds for burn wounds [6]. However, they have poor mechanical characteristics and chemical resistance [15]. Alginate-based dressing promotes wound healing by providing a moist environment, aids in debridement, and prevents trauma to the wound bed and surrounding skin [121]. Due to their high water content, flexibility, permeability, and capacity to generate a moist environment in the wound bed, alginate hydrogels are frequently utilized to treat various wound types [122]. The efficacy of alginate hydrogels can be improved by adding other types of materials, for example, silver, which increases antimicrobial activity and enhances the antioxidant capacity [123]. Similarly, cellulose has also been synthesized into hydrogels for wound treatment. Cellulose-based hydrogels possess high mechanical strength, biocompatibility, biodegradability, and environmental friendliness [124]. They also have the ability to enhance the healing process by retaining and releasing multiple growth factors at the injury site, which can increase cutaneous fibroblast migration and proliferation [6]. However, cellulose does not contain antimicrobial activity to prevent wound infection [125].

Synthetic polymers like polyvinyl alcohol (PVA), polyethylene glycol (PEG), and polylactic co-glycolic acid (PLGA) are well known for their hydrogel properties. In tissue engineering, PVA would be widely used to repair damaged organs or tissues. Using the optimal PVA to water ratio can result in a suitable hydrogel that mimics the properties of natural tissues [15,126]. However, due to the insufficient elasticity, stiff membrane, and partial hydrophilic properties of PVA hydrogel, it cannot be used alone as a wound treatment polymeric membrane [113]. PEG-based hydrogel has been reported to improve and accelerate wound closure. PEG-based hydrogel is a biodegradable adhesive with simple handling procedures, excellent tissue adherence, controlled degradation, and elastomeric mechanical properties, which indicates it is promising as a tissue adhesive sealant for wound care management [127]. PLGA has the benefit of sustained and prolonged drug release, which could be used in drug delivery systems. Although PLGA is a biodegradable polymer, microenvironmental acidity caused by polymer degradation can irritate at the site of formulation application and damage peptide or protein drugs loaded into hydrogels [126].

### 4.2. Research on Hydrogel Incorporated with Apis Honey Types for Wound Healing

Honey-based hydrogel is a feasible and effective strategy for promoting cutaneous wound healing by regulating various events that are critical to the healing process. Data on the physical properties of honey hydrogel demonstrates that the incorporation of honey into the hydrogel system affects the hydrogel matrix [7]. When honey is introduced into a hydrogel matrix, it can have a favorable effect on wound healing such as regulating cell adhesion, spreading, migration, and proliferation [128]. Honey hydrogel dressings possess remarkable physical qualities, including high transparency and the ability to absorb exudates, as well as an acidic pH value, which meets several of the critical characteristics of an ideal wound dressing [7]. As a result, honey hydrogel dressing is a promising new dressing for wound treatment, opening up new options for future study and application.

In a study of honey-loaded PVA/chitosan/montmorillonite/honey responsive nanocomposite hydrogel (PCMH), the in vivo results revealed that PCMH hydrogel samples had more than 99% antibacterial activity due to the presence of honey, which boosted the antibacterial activity. The honey was fully released in the wound bed, restricting microbe development, and shortening the wound healing process through the growth and proliferation of wounded tissue cells [15]. In addition, carboxymethyl cellulose (CMC) hydrogel with chestnut honey (CH) demonstrates an excellent capacity for suppressing and killing bacteria on wound healing. The ability of a CH–CMC hydrogel to retain moisture rises as more chestnut honey is administered. The CH–CMC hydrogel can provide a moist environment for wound healing that lasts at least two days at body temperature. It is also non-adherent, can reach narrow and deep openings, and does not injure living tissue or skin surrounding the wound. Furthermore, it can absorb debris and excretion from inflamed wounds, and can be conveniently removed. However, there is no significant change in the thickness of the granulation tissue [129].

Another study by Fathollahipour et al. [120] showed that PVA/honey hydrogel loaded with erythromycin (PVA-H-E) as a hydrogel sheet was effective and practical for wound dressings. The presence of honey enhances antibiotic diffusion through the hydrogel; the rate of erythromycin release from the PVA-H-E hydrogel is the greatest among other hydrogel samples and decreases in later stages compared to others. PVA-H-E hydrogel also had the most potent adhesive properties and showed a considerably greater inhibitory zone. This finding indicates that the presence of honey enhances antibiotic diffusion through the hydrogel. A study by Tavakoli and Tang [16] developed a honey/PVA hybrid hydrogel for wound dressing with a high concentration of Manuka honey in the presence of varying percentages of borax as a crosslinking agent. The results show that the honey/PVA hybrid hydrogel with a high concentration of honey did not result in antibiotic burst release. The use of borax as a crosslinking agent did not affect the cytotoxicity of hydrogel or their antibacterial effectiveness against wound infection. However, the honey/PVA hybrid hydrogel displayed the highest swellability and stability among the samples, as well as excellent antibacterial activity.

In another study, Mukhopadhyay et al. [130] reported that fabricating a sodium alginate hydrogel coupled with honey (HSAG) via dual crosslinking (ionically and covalently) provided an intermediate stiffness in the fabric and a consistent swelling property and limited erratic degradation of the polymer, thereby providing a conducive environment for cellular growth and proliferation for wound healing. The HSAG hydrogel shows improvement on cell morphology and adhesion. However, the alginate’s hydrophilic surface’s inadequate absorption of serum proteins caused a decrease in cell attachment. The in vivo wound healing study revealed that wounds treated with HSAG healed more quickly and within a shorter period (4 days) than SAG-treated wounds. This demonstrated that honey and alginate had a synergistic impact on wound healing. The HSAG hydrogel is also more effective than traditional fibrotic healing for regenerative wound repair with dermal reconstruction, granulation tissue development, and re-epithelialization.

El-Kased et al. [86] developed and compared the healing and antibacterial capabilities of honey-chitosan and honey-carbopol 934 hydrogels in wound healing. The results demonstrated that honey-chitosan hydrogels had a larger inhibition zone than honey-carbopol hydrogels and exhibited better antibacterial activity. The epidermis and dermis also displayed regeneration, with hyperkeratosis and acanthosis replacing the necrosed skin of the epidermis. However, the increase in honey content resulted in a lower percentage of swelling in the hydrogel. This can be regulated by the polymer type and viscosity, the degree of crosslinking, ionic strength, and the presence of water, all of which significantly impact the swelling process. Moreover, increasing the crosslinker concentration negatively influences the hydrogel swelling index as the structure becomes tighter.

Another study by Giusto et al. [131] demonstrated that a pectin-honey hydrogel (PHH) wound membrane can accelerate the healing process. The in vivo experiments showed that all PHH-treated wounds exhibited a well-developed dermis. The presence of hair follicles and developed fibrous tissue indicated that the wounds had healed effectively. The results suggest that administering PHH promotes wound healing in rats. The advantages of PHH over other hydrogels or honey-based devices are its low cost, ease of manufacture, and ease of application on the wound. This should allow honey membrane wound dressings to be used in economically deprived areas. The findings indicate that the usage of PHH promotes and accelerates wound healing.

### 4.3. Evaluation of Hydrogel Incorporate with Stingless Bee Honey for Wound Healing

Gopal et al. [17] recently performed a study to evaluate the effects of using several kinds of Southeast Asian honey on wounds using cellulose-based hydrogels containing giant bee honey, Asian bee honey, and stingless bee honey. According to the findings, giant bee honey hydrogels show more *S. aureus* and *E. coli* inhibiting zones. This is due to the antibacterial components, such as phenolic acids and flavonoids, found in giant bee honey more prevalently than in stingless bee honey and Asian bee honey. When Asian bee honey hydrogels were compared to stingless bee honey hydrogels, there were higher inhibition zones for *E. coli* but smaller inhibition for *S. aureus.* The phenolic compounds and acidic pH were the non-peroxide antibacterial properties of stingless bee honey that contributed to the antimicrobial agents of the stingless bee honey. The stingless bee honey hydrogels also showed the maximum cell viability and cell migration at the wounded area.

Another study by Abd Jalil [18] on the development of PVA-natural biopolymer hydrogel, incorporated with stingless bee honey, found that the honey hydrogel exhibited excellent swelling properties, providing an excellent moisturizing environment to the wound. It also exhibited excellent antimicrobial properties and was free from pathogenic microorganisms. An in vivo trial demonstrated that honey hydrogel therapy led to much faster healing in the group treated than in the no-treatment group. The amount of collagen deposited was abundant in the honey hydrogel treated group, and the structure of the fibroblast was well organized. This indicated that the wound entered the remodeling phase at the end of the animal study compared with the no-treatment group, which was still in the proliferation phase.

To ensure the alginate wound dressing containing stingless bee honey from the *Tetragonula biroi* does not become contaminated, a study by Baldos et al. [132] investigated the effect of radiation sterilization on this particular alginate wound dressing. The stingless bee honey alginate wound dressing (HAWD) can be highly permeable and is suitable for heavy exudate wounds. The HAWD dressings were exposed to a sterilization dose of 25 kGy, which is safe enough for health care use. These data reveal that electron beam irradiation had no effect on the pH, total soluble solids, and flavonoids but did increase the total phenolics in stingless bee honey. The required irradiation dose of 25 kGy was able to remove microbial contamination in the HAWD, and the physicochemical properties of HAWD remained unaltered. Thus, HAWD was successfully sterilized with a dose of 25 kGy. Hence, it has the potential to serve as a natural product-based alternative to commercially available wound care solutions.

Based on the studies reviewed, stingless bee honey has high efficacy in treating wounds as it can prevent infections, promote cell viability, and speed up the wound’s recovery. As for the stingless bee honey-based hydrogel, there are many promising properties such as good hydrophilicity, microbial safety, reliable release profile, and efficacy in the wound healing treatment. The hydrogel combination with stingless bee honey is used because of the difficulty applying honey directly to the wound bed. The honey would flow out of the wound bed as time passed by. This would cause inconvenience for the patient. Consequently, the incorporation of honey in a hydrogel system is more beneficial and applicable. Additionally, a study on the sterilization of stingless bee honey-alginate wound dressing showed it has significant benefits. It is crucial to have a sterile dressing that is safe for wound healing applications. For future recommendations, the development of stingless bee honey-based hydrogel should be convenient for the patient. The dressing should be easy to apply directly to the wound. The stingless bee honey and hydrogel material can be crosslinked at the wound site by photo-crosslinking, which only takes a few seconds to crosslink and completely harden. In this situation, the secondary dressing would not be needed anymore, and this could minimize the pain of dressing changes and avoid skin abrasion. Finally, the future stingless bee honey-based hydrogel should be cost-effective so that everyone can afford to have it.

## 5. Summary

To summarize, stingless bee honey promises significant benefits for healing wounds, such as preventing wound infection, keeping a moist environment, enhancing cell proliferation and viability, reducing pain, and preventing skin abrasion and maceration. This is due to stingless bee honey’s therapeutic properties, including antioxidant, antibacterial, anti-inflammatory, and moisturizing capabilities. In addition, stingless bee honey-based hydrogel also provides good feedback on the wound site, encouraging the proliferation and epithelization of cells and speeding up the wound healing rate. Therefore, it can be concluded that stingless bee honey-based hydrogel has a high potential to be a good wound dressing. Clinical studies of stingless bee honey should be continued in order to produce an excellent wound dressing and to enhance existing wound dressings.

## Data Availability

The data presented in this study are available on request from the corresponding author.
